# Stress Effects on Multiple Memory System Interactions

**DOI:** 10.1155/2016/4932128

**Published:** 2015-12-27

**Authors:** Deborah Ness, Pasquale Calabrese

**Affiliations:** Faculty of Psychology and Interdisciplinary Platform Psychiatry and Psychology, Division of Molecular and Cognitive Neuroscience, Neuropsychology and Behavioural Neurology Unit, University of Basel, Birmannsgasse 8, 4055 Basel, Switzerland

## Abstract

Extensive behavioural, pharmacological, and neurological research reports stress effects on mammalian memory processes. While stress effects on memory quantity have been known for decades, the influence of stress on multiple memory systems and their distinct contributions to the learning process have only recently been described. In this paper, after summarizing the fundamental biological aspects of stress/emotional arousal and recapitulating functionally and anatomically distinct memory systems, we review recent animal and human studies exploring the effects of stress on multiple memory systems. Apart from discussing the interaction between distinct memory systems in stressful situations, we will also outline the fundamental role of the amygdala in mediating such stress effects. Additionally, based on the methods applied in the herein discussed studies, we will discuss how memory translates into behaviour.

## 1. Introduction

In the following, the biological correlates and mechanisms of stress and their influence on memory processing will be discussed in order to outline the basic mechanisms underlying the differential ways in which memory can be affected by a stressor.

### 1.1. The Biology of Stress and Emotional Arousal

Stress refers to an organism's physiological and psychological reaction triggered by an external or internal stressor, such as an environmental condition or a psychological stimulus. It is a state of mental or emotional strain or tension resulting from adverse or demanding circumstances and can last for just a few minutes to hours (acute stress) up to months or even years (chronic stress). Stress is a highly subjective experience in the sense that equal events are not perceived as equally stressful by different individuals. Thus, stress can be caused by numerous diverse events, including hassles of everyday life (e.g., time pressure) and life-threatening situations or circumstances (e.g., war or natural disasters). Whether a situation is experienced as stressful or not is determined by complex interactions between different brain regions, including the prefrontal cortex, the hippocampus, and the amygdala [1, 2]. The involvement of these structures in the process of appraisal is critical to the ability to link the currently experienced situation with one's past experiences in order to modulate adaptive behaviour.

The aforementioned limbic structures as well as the prefrontal cortex have connections to the hypothalamus which plays a crucial role concerning the activation of a physiological stress response induced by endocrinologic changes [2]. There are two main classes of stress hormones: (1) glucocorticoids (GCs; corticosterone in rodents, cortisol in humans) and (2) catecholamines (epinephrine and norepinephrine).

**Figure 1:**
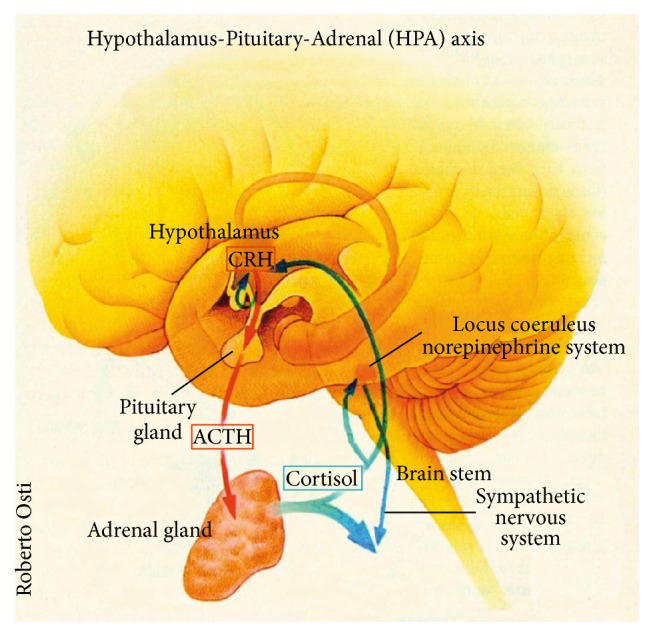
The Hypothalamus-Pituitary-Adrenal (HPA) axis. CRH = corticotropin-releasing hormone; ACTH = adrenocorticotropic hormone [6].

If a situation is perceived as stressful, neurons located in the paraventricular nucleus of the hypothalamus synthesize and release corticotropin-releasing hormone (CRH) which in turn triggers the release of adrenocorticotropic hormone (ACTH) from the pituitary gland into the bloodstream. ACTH acts on the adrenal glands and thus induces the release of GCs from the adrenal cortices. GCs alter the function of multiple body tissues in order to mobilize or store energy to meet the demands of a stress challenge [3]. Through a negative feedback mechanism GCs inhibit CRH as well as ACTH secretion as they bind to GC receptors in the hypothalamus and the hippocampus, hence terminating the stress response when the threatening situation is over [4, 5]. This stress system is known as the hypothalamic-pituitary-adrenal (HPA) axis (Figure 1).

There are two kinds of receptors which are targeted by GCs: (1) the low-affinity mineralocorticoid receptor (MR) and (2) the high-affinity glucocorticoid receptor (GR) [7]. While MRs are almost exclusively expressed in the hippocampal formation, GRs are widely distributed throughout the brain with especially high concentrations in the hippocampus, the amygdala, and the prefrontal cortex. As already mentioned, these brain regions play a critical role in the process of cognitive appraisal and are also actively involved in the negative feedback mechanism regulating HPA axis activation [8]. When there is no current stressor and the body is at rest, MRs are usually occupied, while GC levels are too low to bind to the low-affinity GRs. However, when GC levels rise, such as in the case of (chronic) stress, the GC concentration becomes high enough to activate the low-affinity GRs as well [3].

**Figure 2:**
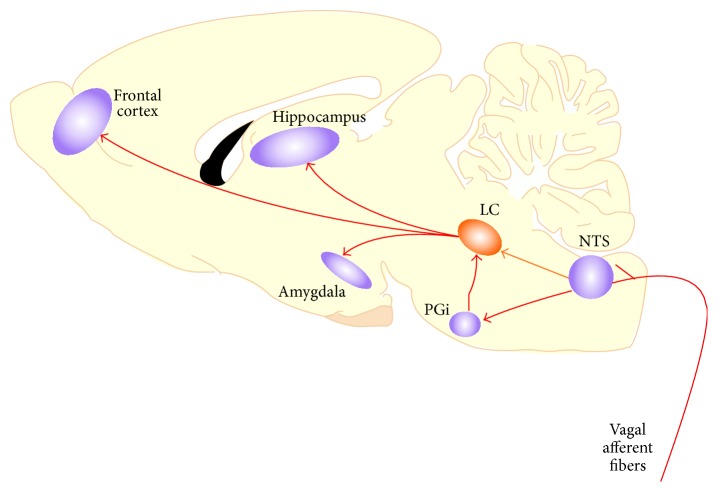
Schematic diagram depicting the activation of central structures through noradrenergic projections from the locus coeruleus (LC) in the rat brain. The nucleus tractus solitarius (NTS) receives peripheral input via the vagus nerve which is activated after stressful or emotionally arousing experiences [9].

Apart from activating the HPA axis and its rather slow hormonal effects, the hypothalamus is also crucial for the activation of the autonomic nervous system in response to emotional arousal, which is produced by either aversive stressors or highly rewarding events [9]. This activation is initiated only seconds after a stressful event, whereas it takes up to 25 minutes until GC levels reach their peak. Autonomic nervous system activation triggers the release of epinephrine and norepinephrine from the adrenal medulla. Due to the characteristics of the blood brain barrier, catecholamines cannot enter the central nervous system, but they can indeed influence its activity through the complex connections between the autonomic and the central nervous system. Higher peripheral epinephrine levels induced by emotional arousal indirectly stimulate the release of central norepinephrine in the basolateral amygdala (BLA; Figure 2). Ascending fibres of the vagus nerve contain adrenergic receptors, which become activated through the binding of peripheral epinephrine in rats [10] and in humans [11]. These fibres transmit information regarding heightened activity in visceral sensory organs to the central nervous system, namely, to the nucleus tractus solitarius (NTS), which is located in the brainstem [12, 13]. In turn, NTS neurons activate the locus coeruleus (LC) through direct synapses and thus influence central norepinephrine activity [14]. Since most of the noradrenergic terminals in the BLA originate in the LC, the release of norepinephrine from the BLA seems to be mainly influenced by the activity of the LC [15–18]. Apart from its influence on the BLA, the LC also has extensive connections to the hippocampus and the prefrontal cortex, regions which are critically involved in memory processes (Figure 2). Norepinephrine is one of the primary neurotransmitters mediating the communication between these structures.

Apart from the above-described central effects of stress and emotional arousal, other sympathetic reactions, like elevated heart rate, blood pressure, and galvanic skin response, are also consistently reported [19–25]. Moreover, many other hormones, neurotransmitters, and neuropeptides are released after stressful experiences, which helps the organism to successfully adapt to the stressor and restore homeostasis [9].

Moreover, it has been proposed that increased plasticity provided by developing neurons in the hippocampus may increase an individual's capacity to adapt to a changing environment [26]. Recently, it has been theorized that the hippocampal formation, which is crucial to memory formation as well as spatial navigation, might also play an important role in stress regulation, possibly through the regulation of adult neurogenesis. For example, it has been found that artificially reduced neurogenesis through transgenic modifications or radiation exposure leads to an increased level of stress hormones following a stressful experience [27, 28]. This indicates that adult neurogenesis may be able to enhance the GC-mediated negative feedback mechanism of the HPA axis and thus could eventually act as a buffer to stress. In addition, GCs acting on GRs can result in the modulation of gene transcription through several complex molecular pathways, some of which are also involved in neurogenesis, namely, the forkhead box protein O3 (FOXO3A) pathway, which is activated, and the transforming growth factor *β* (TGF*β*)-SMAD2-SMAD3 pathway and the Hedgehog pathway, which are inhibited [29–34].

Moreover, studies could show that stress also initiates the release of proinflammatory cytokines in the hippocampus and several other brain regions, where interleukin 1*β* levels are increased through a catecholamine-mediated mechanism [35]. Interestingly, stress also influences several neurotrophic factors, which are important for the growth and maintenance of neurons and thus their proper functioning. Among the neurotrophic factors sensible to stress are brain-derived neurotrophic factor (BDNF), vascular endothelial growth factor (VEGF), and neuregulin 1 (NRG1), which is part of the epidermal growth factor family of proteins [36].

However, we are still far from an integrative, complete understanding of the many effects stress has on a molecular level, especially when it comes to linking the knowledge to complex cognitive phenomena (e.g., memory) or even behavior. Since the neurobiological understanding of stress on a microlevel is still in its infancy and because it is beyond the scope of this review, we will focus on the above-described general biological stress response systems (HPA axis and autonomic nervous system), which are essential to understand the studies to be discussed in our review. However, it is of great importance to acknowledge that the correlates we describe in this paper (e.g., brain activity patterns) to explain the according behavioural findings* are themselves* the result of multiple complex interactions of different molecular pathways within each cell.

### 1.2. The Neuroanatomy of Multiple Memory Systems

Besides a chronological framework of memory processing (encoding, consolidation, and retrieval) a content-based subdivision of memory has been introduced and scientists became realerted to terms like conscious and nonconscious information processing. Whereas conscious memory processes are part of the declarative memory system, nonconscious information processes belong to the nondeclarative system. Since declarative memory allows for encoding the relationships between multiple items and events, it is considered to be representational, hence providing an internal model of the external world which is either true or false. Stored representations are highly flexible and thus able to guide performance in many different situations occurring in a changing environment [37]. In contrast, nondeclarative memory contents are not subject to conscious recollection, are not representational, and are thus neither true nor false. An important ability regarding nondeclarative memory processes lies in the extraction of common elements from a series of events. Nondeclarative memory formation can be described as modification of specialized performance systems, which become reactivated in situations similar to the original learning context [37].

**Figure 3:**
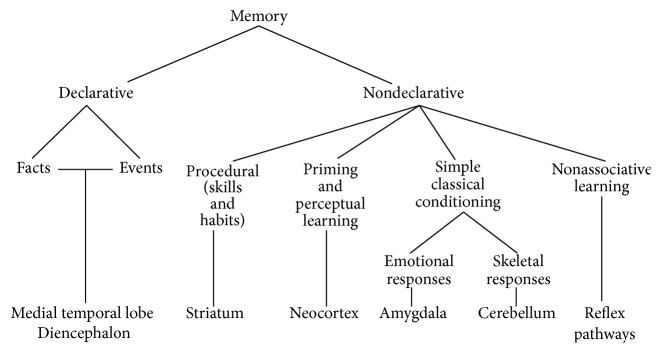
Taxonomy of multiple memory systems [37].

Declarative memory is further subdivided into semantic and episodic memory (Figure 3). While episodic memory means context-based information processing with the possibility of “travelling back in time” and hence integrating a memory in its original spatiotemporal context, semantic memory (knowledge) is context-free [38]. Both of these declarative memory systems critically rely on brain structures in the medial temporal lobe (e.g., hippocampus) and the diencephalon. Additionally, other brain structures, such as the prefrontal lobe, also participate in episodic memory processes [39, 40].

Nondeclarative memory is further divided into four subsystems, which are responsible for functionally distinct processes: (1) procedural/habit memory, which refers to skill-based and largely automatic processes and is dependent on the striatum, (2) priming/perceptual learning—the phenomenon of an increased likelihood of reidentifying a previously subconsciously perceived stimulus/item—which is regulated by the neocortex, (3) conditioning, which involves the amygdala and the cerebellum, and (4) nonassociative learning, which operates over reflex pathways (Figure 3).

The described memory systems are simultaneously engaged in the parallel processing of information and can operate in a competitive or cooperative manner [41]. Seminal studies were able to show that memory operations in the mammalian brain do operate via anatomically distinct systems. In a study of Packard and coworkers [42] rats with fimbria-fornix lesions were impaired in a win-shift paradigm of the radial-maze test and caudate animals were unimpaired relative to controls. Conversely, rats with fimbria-fornix lesions were superior to controls in choice accuracy on the win-stay version radial-maze task, while caudate animals were impaired relative to control animals. This double dissociation indicated a different contribution of multiple memory systems which can be probed by different tests: (1) spatial navigation, (2) probabilistic classification learning (PCL), and (3) instrumental learning.

**Figure 4:**
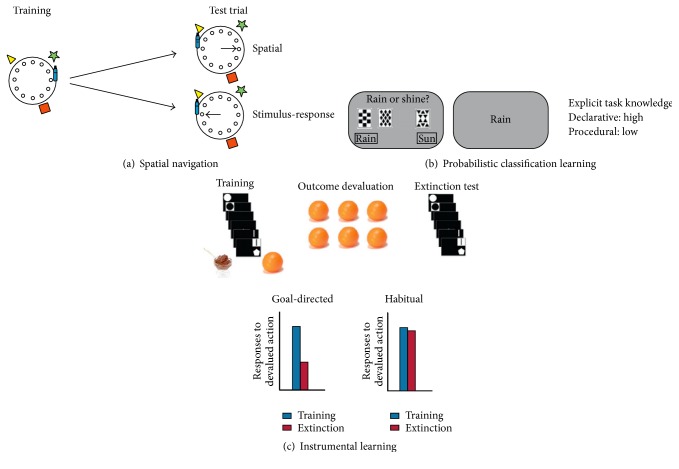
Behavioural tests to distinguish between multiple memory systems: (a) spatial navigation, (b) probabilistic classification learning, and (c) instrumental learning [2].

Spatial navigation tasks use single proximal as well as multiple distal cues, which help the subject to respond (Figure 4(a)). The hippocampal spatial memory system creates a cognitive map by associating multiple cues. In contrast, the procedural stimulus-response (S-R) memory system learns the association between a proximal cue/stimulus and a response. The S-R memory system is dependent on the dorsal striatum [2]. When subjects are trained in a spatial navigation task, subsequent behaviour in a test, where only the proximal cue is relocated, differs according to the used memory system. Going to the relocated proximal cue indicates S-R learning, whereas going to the originally reinforced target indicates multiple cue spatial learning.

In PCL tasks (e.g., the weather prediction task), subjects are trained in categorizing different stimuli (multiple cues on one to three out of four cards) to predict an outcome (sun versus rain) based on trial-by-trial feedback (Figure 4(b)). When learning has been controlled by the hippocampus-dependent declarative system during training, the explicit knowledge of the task is expected to be higher than after using the striatum-based procedural system.

Instrumental learning works with the paradigm of outcome devaluation (Figure 4(c)). Subjects are trained in two instrumental actions leading to distinct food rewards, one of which is subsequently devalued by unlimited access to this kind of food. If subjects use a goal-directed learning strategy dependent on the prefrontal cortex, they favour the food outcome which has not been devalued over the devalued food in a subsequent extinction test. A goal-directed learning strategy encodes the relationship between action and outcome and thus makes subjects sensitive to changes regarding the outcome value of their actions. In contrast, the lack of such behaviour indicates dorsolateral striatum-dependent S-R learning and is referred to as habit learning, where the relationship between the stimulus and the response is encoded without a link to the actual outcome [2].

### 1.3. Memory Formation under Stress

The brain regions involved in a physiological stress response extensively overlap with the structures, which are critical for memory processes. A great amount of evidence from numerous studies confirms that stress and emotional arousal affect memory. In the following, the effects of stress on quantitative mostly time-dependent aspects and on qualitative aspects which involve multiple memory systems will be reported.

#### 1.3.1. Stress Effects on Quantitative Memory Performance

Stress affects the amount of memory, that is,* how much* an individual remembers. These stress effects have been extensively studied with particular focus on hippocampus-dependent memory processing, showing time-dependent effects of the stressor on encoding, consolidation, and retrieval [8, 43]. The effects of stress on encoding are difficult to assess because they are always confounded with consolidation and retrieval processes. It is probably this difficulty which led to the highly conflicting results of different researchers studying stress effects on encoding. While some studies indicate enhancing effects of stress on memory encoding [44, 45], others reported impairing effects [46–49]. However, some of these contradictions might be due to differences in emotionality, which has been shown to critically influence memory processes. In a state of higher central norepinephrine triggered by emotional arousal, memory for negative information (e.g., negative pictures) appears to be enhanced in the presence of GCs. At the same time though, memory for neutral information seems to be impaired [50, 51].

**Figure 5:**
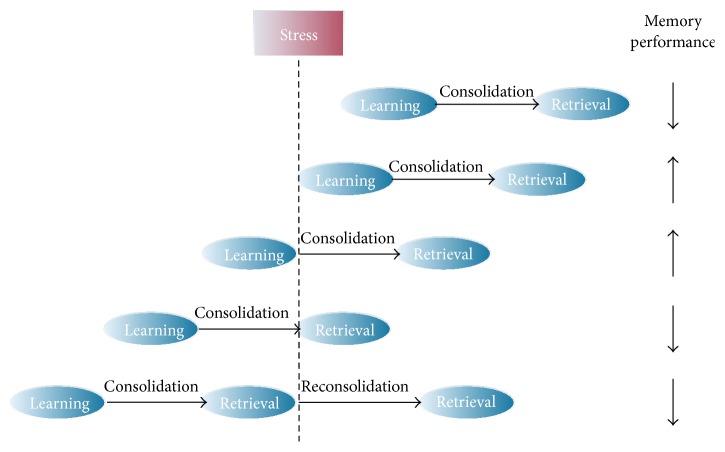
Stress affects memory performance in a different manner dependent on the timing of the stressor [2].

The adrenal hormones released by emotional arousal thus seem to be important regulators of memory strength: the consolidation enhancing effects of GCs disappear without the cooccurrence of norepinephrine in the BLA. In line with this, lesions of the BLA or the administration of beta-adrenergic receptor antagonists in the BLA abolish the enhancing effects of high GC levels on memory consolidation [52, 53]. Furthermore, it has been shown that GR antagonists impair, whereas GCs enhance consolidation of emotionally relevant information [54–56]. This enhancement of the consolidation processes induced by stress hormones is also well supported in human studies [57]. In contrast, stress impairs retrieval of previously learned spatial material. Spatial memory in rodents is severely impaired when they are stressed or received GCs before retention testing [58, 59]. However, beta-adrenergic receptor antagonists or a lesion in the BLA abolishes the impairing effects of GCs on spatial memory retrieval [60, 61]. Thus, the effects of stress on memory retrieval also underlie the influence of noradrenergic activity in the BLA. This is also the case in humans: stress- or pharmacologically induced increases in GC levels do not impair memory retrieval in a nonarousing situation [62] or after blockage of the beta-adrenergic receptors in the BLA [63, 64]. It is important to recognize that there are also some studies which report contradicting results [65–67]. For example, Domes and colleagues [66] detected no global effect of cortisol on neither verbal nor nonverbal memory. However, it became evident that stress effects on memory depend on (1) the timing of the stress response as well as (2) the convergence of stress hormone activity [43, 68, 69]. In general, a physiological stress response is beneficial when occurring during the learning episode but impairs memory function when experienced during retrieval (Figure 5).

#### 1.3.2. Stress Effects on Memory Quality

Apart from the stress effects on quantitative aspects of the memory, stress and emotional arousal also affect the processing mode. Rodents stressed before training in a spatial navigation task used the striatum-dependent S-R strategy more often than hippocampus-dependent spatial strategies in the test trial [47]. Moreover, anxiogenic drugs which induce strong emotional arousal also facilitate the use of S-R strategies and reduce the use of spatial strategies [70, 71]. In humans, stress before training in a spatial navigation task also favoured the use of an S-R strategy over a spatial strategy [72]. Interestingly, according to the aforementioned studies, the adopted learning strategy does not necessarily influence the quantitative memory performance.

Moreover, when participants are stressed in an instrumental learning task, they are biased towards the use of a habitual strategy, which renders them insensitive to changes in the outcome value. In instrumental learning tasks as well as in spatial navigation tasks, stress prior to training appears to favour striatum-dependent S-R learning strategies over prefrontal cortex-dependent goal-directed or hippocampus-dependent spatial strategies, respectively [73].

There is evidence suggesting that chronic stress can have similar effects on the use of multiple memory systems. Like under acute stress, chronically stressed rodents favour the use of a habit strategy in spatial navigation [74] and instrumental responding [75]. Moreover, an enlargement of the dorsolateral striatum as well as medial prefrontal cortex-atrophy is associated with the switch from goal-directed to habit learning in chronically stressed rats [75]. An enlargement of the amygdala has also been reported in association with chronic stress [76], which is likely to also have an effect on the usage of multiple memory systems.

## 2. Comparison of Selected Studies

As mentioned before, stress effects on memory are evident for qualitative as well as for quantitative aspects of memory and their nature may be time-dependent. Stress effects on the engagement of multiple memory systems seem to favour striatum-dependent learning strategies and to render behaviour habitual. The studies discussed in this section will further address the question of whether the enhancement of striatum-based habit memory may be the result of an impaired hippocampus-based cognitive memory.

Moreover, in regard to the crucial role of the amygdala in the modulation of quantitative memory parameters under stress, the contribution of this brain region to the engagement of multiple memory systems is of interest. Studies investigating the role of the BLA in the switch from cognitive to habit memory will therefore also be included in this section.

The above-described tests for the study of multiple memory systems assess active behavioural choices. Therefore, the question of how memory translates into behaviour will also be discussed.

### 2.1. Competition of Multiple Memory Systems

In order to study which memory system or which learning strategy* is favoured* under stress, a classical spatial navigation task can be used. However, such a dual-solution task cannot be used to investigate the influence stress has on habit and cognitive memory separately. To be able to explore the potentially distinct effects stress may have on hippocampal cognitive and striatal habit memory, two single-solution tasks are required. Both of the studies conducted by Wingard and Packard [77] and Packard and Gabriele [78] made use of this advantage of applying two single-solution tasks. In both experiments, adult male rats were trained in a water-maze plus task which required either the use of cognitive “place” or habitual “response” learning. Rats trained in the place learning version always had to go to the same arm of the maze (e.g., the west arm) independently of the start position to reach the escape platform, which reinforced the use of a spatial strategy. In contrast, in the habit learning version of the maze, only a turn in the same direction leads to the escape platform, which reinforced the use of a habit strategy.

**Figure 6:**
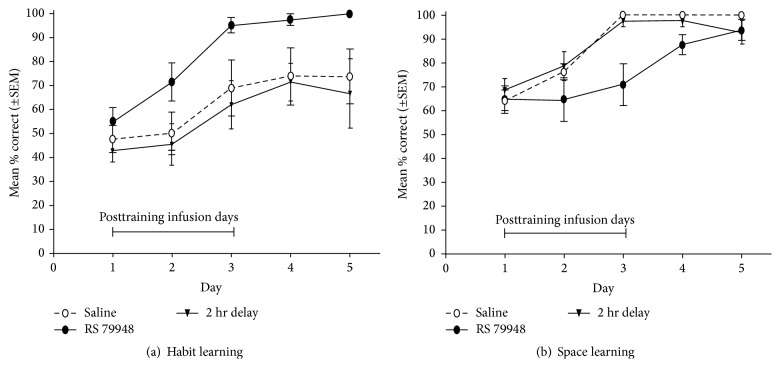
Intra-BLA infusions of the beta-adrenergic antagonist RS 79948 (a) enhance the acquisition of habit learning and (b) impair the acquisition of space learning strategies in comparison to a control condition (saline) and a 2 h delayed administration of RS 79948 [77].

In the study by Wingard and Packard [77], a physiological stress response was induced after training by injections of a beta-adrenergic antagonist in the BLA, which leads to an increase of central norepinephrine levels. As discussed earlier, a rise in central norepinephrine levels is a hallmark of the physiological effects triggered by stressful or emotionally arousing experiences. The effects of this pharmacologically induced stress response on the acquisition of either space or habit learning strategies is revealing: while performance in the place learning version was significantly impaired, performance in the habit learning version was dramatically enhanced under stress compared to the control condition (Figure 6). Interestingly, whereas these effects were present after an immediate infusion of the drug after learning, a 2 h delayed infusion remained ineffective [77]. These results suggest that higher norepinephrine levels due to stress or emotional arousal seem to time-dependently affect consolidation processes of multiple memory systems.

In a subsequent study [78], these results could be replicated using peripheral injections of RS 79948 instead of intra-BLA infusions of the drug. How peripheral stress hormone levels can affect central noradrenergic activity has been described previously. These pathways elegantly explain why peripheral administration of the drug results in similar central activity patterns as intra-BLA infusions and is thus also able to distinctly affect consolidation processes of multiple memory systems. The findings suggest that the switch to striatum-dependent habit learning strategies under stress in classical dual-solution tasks appears to be a result of both enhanced habit memory and impaired cognitive memory.

**Figure 7:**
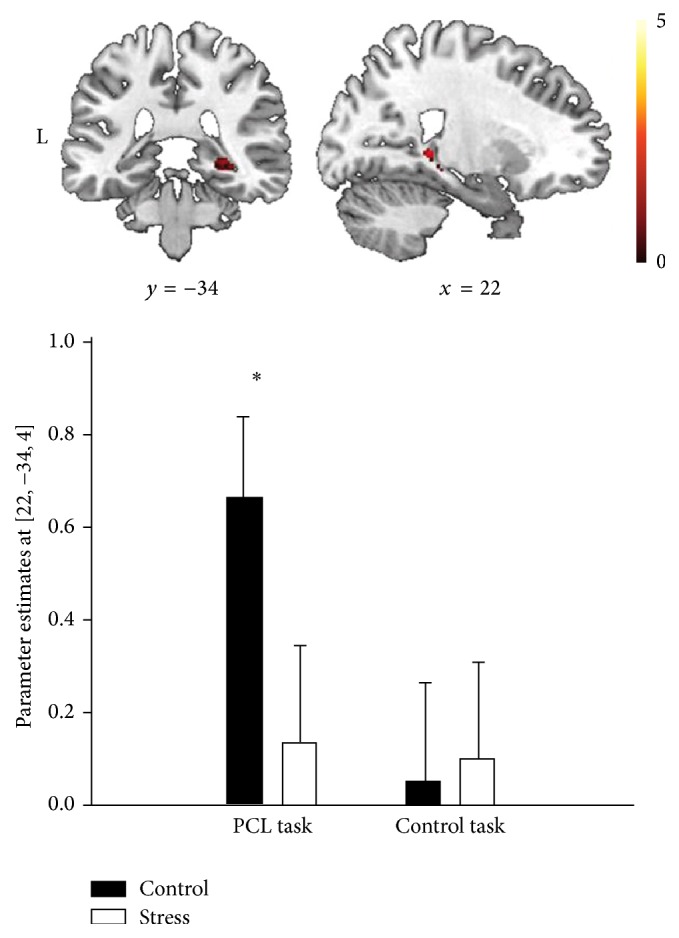
Neuroimaging data assessed during PCL shows less right hippocampal activity during PCL in the stress group compared to the control group [79].

Additional evidence regarding this idea comes from a study using functional magnetic resonance imaging (fMRI) by Schwabe and Wolf [79], where 60 healthy participants solved a PCL task in a scanner. Participants who were stressed with the socially evaluated cold pressure test (SECPT) before the PCL task were biased towards the use of a striatum-dependent multicue learning strategy at the expense of a hippocampus-dependent single-cue learning strategy. As expected, using multicue learning strategies was correlated with more activity in the neostriatum (putamen and caudate nucleus), while the use of single-cue learning strategies has been associated with increased activity in the hippocampal formation [79]. Neuroimaging data revealed further that the striatum was significantly activated during PCL in both groups, but that there was no significant activation of medial temporal lobe structures in the stress group (Figure 7). This is in line with the idea that the hippocampal declarative memory system is impaired under stress.

**Figure 8:**
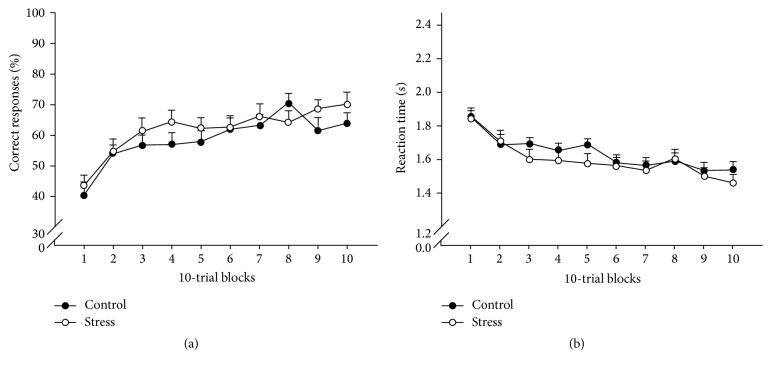
Comparable learning curves in the PCL task of the stress group and the control group: gradual improvement of classification performance across training. (a) Percentage of correct responses and (b) reaction time in seconds [79].

Interestingly, PCL performance in the control group was correlated with activity in the left hippocampus but not with striatal activity. In contrast, PCL performance in the stress group was positively correlated with activity in the right caudate nucleus and the left putamen, but negatively correlated with activity in the left hippocampus [79]. However, the learning curves of stress and control groups were comparable: over time an increase of the percentage of correct responses and a decrease of reaction time were observed across groups (Figure 8). This indicates that stress is not hindering the acquisition of the PCL task but rather changes the applied learning strategy from hippocampus-dependent declarative to striatum-dependant procedural learning.

These data demonstrate how a shift from cognitive to habit memory systems under stress can rescue task performance. Because the hippocampal memory system is impaired under stress its inhibitory or competitive effects on the striatal memory system are reduced or abolished. Importantly, the negative correlation between left hippocampal activity and task performance after stress suggests that attempts to engage the declarative memory system during a stressful experience even disrupts task performance [79]. Thus, cognitive and habit memory systems seem to interact in a primarily competitive manner.

### 2.2. The Role of the Amygdala

As mentioned beforehand, in the study conducted by Wingard and Packard [77], infusions of the beta-adrenergic receptor antagonist RS 79948 right into the BLA impaired hippocampus-dependent memory and enhanced striatum-dependent memory in rodents. Thus, the amygdala seems to play a crucial role when it comes to the modulation of multiple memory systems. The rise of central norepinephrine levels may affect the efferent projections of the BLA in a manner that impairs synaptic plasticity in the hippocampus, which consequently results in impaired cognitive memory.

**Figure 9:**
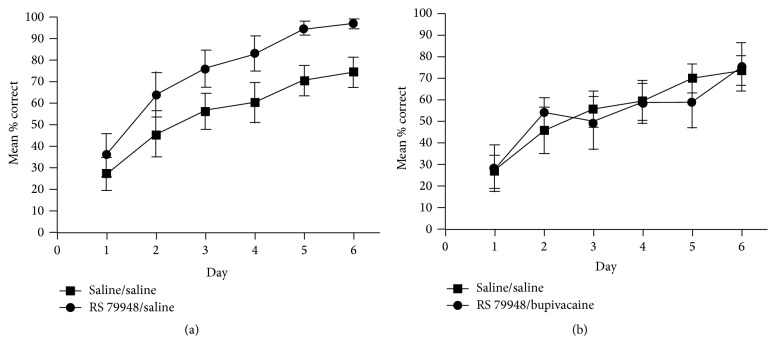
(a) Posttraining peripheral injections of RS 7948 enhance habit memory, but (b) BLA inactivation with bupivacaine blocks this enhancement [78].

**Figure 10:**
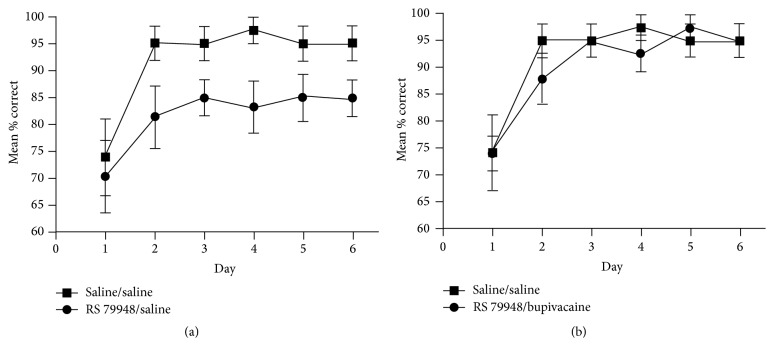
(a) Posttraining peripheral injections of RS 7948 impair cognitive memory, but (b) BLA inactivation with bupivacaine blocks this impairment [78].

The study by Packard and Gabriele [78] further investigated whether the functional integrity of the BLA is necessary in order for norepinephrine levels to affect the engagement of cognitive and habit memory. Therefore, the functional integrity of the BLA was disrupted using direct injections of the sodium channel blocker bupivacaine. As previously mentioned, posttraining peripheral injections of RS 79948 enhanced response learning and impaired place learning [78]. Importantly, these enhancing and impairing effects were abolished when the BLA had been inactivated (Figures 9 and 10).

Importantly, Packard and Gabriele [78] also showed in their experiment that an inactivation of the BLA without peripheral injections of RS 79948 affected neither place nor response learning. This means that the functional integrity of the BLA is not necessary for the acquisition of either learning strategy. However, the impairing and enhancing effects of peripheral stress hormone levels on hippocampus-dependent cognitive and striatum-dependent habit memory, respectively, require an intact BLA.

Although Schwabe and Wolf [79] did not find significant differences regarding the activation of the amygdala during the PCL and the visuomotor control task for the stress and the control group, the authors suggest that the enhancing and impairing effects of SECPT-induced stress on habit and cognitive memory could nevertheless be mediated by the amygdala. Considering this possibility, a reasonable explanation of the findings might be that the mere fact that the task had to be performed in a scanner could have been arousing to some extent, leading to enough activity in the amygdala in order for the SECPT-induced stress hormones to unfold their effects.

The role of the amygdala in emotionally arousing situations has been described earlier in this paper. Regarding the results of the herein discussed studies, it can thus be concluded that the emotional state can modulate the degree of interference between cognitive and habit memory systems and even release habit memory from competing/inhibitory influences of cognitive memory.

### 2.3. How Memory Translates into Behaviour

Free recall or recognition tasks, which are often used to study quantitative parameters of memory, directly inspect an individual's mere* ability to remember* previously learned, mostly declarative material. On the other hand, the tests used in studies examining qualitative aspects of memory assess an individual's* behavioural choices* in order to clarify which memory system is engaged in a task. This is especially clear in the case of instrumental learning tasks, where participants are trained in two instrumental actions, as described above. Because the engagement of different memory systems is able to modulate an individual's choice of a particular action, we will subsequently discuss how memory may translate into behaviour.

**Figure 11:**
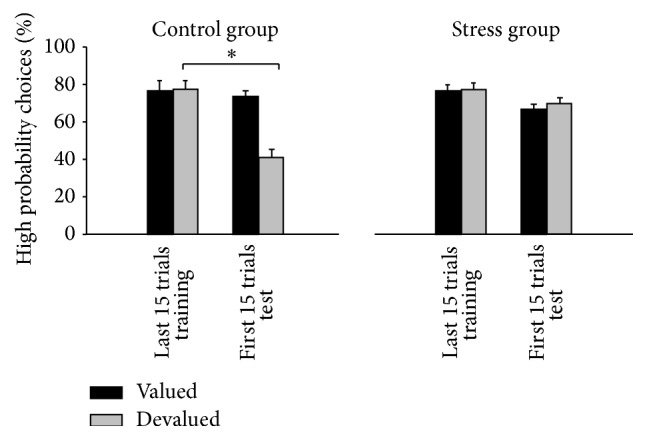
Percent of high probability actions of controls and stressed participants in the last 15-trial block of training and the first 15-trial block of extinction testing [80].

In a study conducted by Schwabe and Wolf [80], participants were trained in two instrumental actions leading to two different food rewards (chocolate/oranges). One of the instrumental actions led to a high probability of receiving a particular food reward, while the other instrumental action never led to the rewarding food outcome but instead induced a low probability of receiving a common outcome (peppermint tea). After the training session, participants were invited to eat one of the rewarding foods to satiety in order to devalue this particular food outcome. Participants underwent a SECPT or control procedure before the subsequent extinction test, where only the common outcome was delivered. Hunger and pleasantness ratings revealed that devaluation was successful across groups because both the stress group and the control group ranked the pleasantness of the devalued food significantly lower before the extinction test [80]. However, while control participants chose the high probability action of the devalued food outcome significantly less often during the extinction test than during training, this behaviour was not observed in stressed participants (Figure 11). These results indicate the use of the dorsal-striatum-dependent habit memory after an acute stressor. They are in alignment with the studies by Wingard and Packard [77] and Packard and Gabriele [78] which both reported enhanced response learning after stress, as well as with the fMRI study by Schwabe and Wolf [79], which found a correlation between such procedural learning processes and striatal activity in a PCL task.

In the experiment by Schwabe and Wolf [80], the stress-induced use of the habit system thus made participant's behaviour insensitive to changes in outcome value. It is of utter importance to notice that the relative engagement of multiple memory systems during encoding and consolidation processes has not been stress-manipulated in this study [80]. The originally used learning strategy can therefore be at least partly neglected when retrieval processes are sufficiently affected by stress.

Since the quality of retrieved information plays an important role in the cognitive appraisal of a situation, stress prior to retrieval can influence subsequent behavioural choices. In the instrumental learning task used by Schwabe and Wolf [80], the retrieved information under stress was dependent on the dorsolateral striatum-based habit memory, which contains information about the S-R relationship but is ignorant of the outcome value of an action. Therefore, the behaviour of stressed participants in the extinction test did not reflect participant's decreased pleasantness rankings of the devalued outcome [80]. Participant's behavioural choices were thus affected by stress effects on the quality of the retrieved information.

In the control condition on the other hand, participant's behavioural choices were congruent with the value they associated to a particular outcome [80]. This goal-directed behaviour indicates that the retrieved information in the extinction test contains sufficient knowledge about the task to establish action-outcome relationships and thus allows the individual to accordingly adapt behavioural choices. However, the assessment of explicit task knowledge after the extinction test revealed no significant differences between the stress and the control groups [80]. This may at first seem to contradict the idea that procedural memory only contains information about the relationship between a stimulus and a response. Interestingly, explicit task knowledge was examined after the extinction test, when salivary cortisol levels of the stress and the control groups were comparable [80]. Thus, it can be assumed that, during the test assessing task knowledge, participants of the control group as well as of the stress group were able to retrieve information concerning the action-outcome relationship. During the extinction test, however, this information was accessible only to the control group, but not to the stress group due to stressed participant's higher cortisol levels.

This is in line with the findings concerning task knowledge acquisition reported in the fMRI study by Schwabe and Wolf [79]. In this study, the authors could show that stress previous to a PCL task reduced participant's explicit knowledge of the task. Cortisol levels at the time of examination of task knowledge were not significantly different in the stress and the control groups. Taken together, these results suggest that the stress-induced enhancement of the striatum-dependent procedural memory system during encoding or consolidation process renders subsequent behavioural choices habitual because only S-R relationships have been learned and can thus be retrieved. Interestingly, stress solely affecting retrieval processes also makes behaviour habitual [80]. This suggests that the quality of retrieved information which is used to make behavioural choices, critically depends on (1) the primarily engaged memory system during the learning process and (2) the memory system engaged in retrieval. It seems that during encoding and consolidation at resting-state both memory systems separately and simultaneously store information, but the subsequent access to either of these systems and its information is affected by an individual's emotional state during retrieval.

## 3. Discussion

An overwhelming number of studies have reported stress effects on memory formation. This evidence can be explained regarding the fact that the brain regions involved in a physiological stress response extensively overlap with the structures which are critical for memory processes. A stressful or emotionally arousing experience can (1) activate the HPA axis which results in rising GC levels and (2) lead to an increase in central norepinephrine levels. There is ample evidence that these stress hormones interact in the BLA, which then distinctly affects memory processes in other regions of the brain, such as the hippocampus [53, 81]. For example, stress can enhance memory consolidation, particularly for emotionally relevant information, as well as impair memory retrieval in both rodents and humans [54–59, 62–64].

Moreover, stress modulates the engagement of multiple memory systems and favours striatum-dependent habit memory over hippocampus-dependent cognitive memory in rodents and humans, without necessarily affecting task performance in a spatial navigation task [47, 70, 72]. Additional evidence suggests that stress also favours the striatum-dependent habit system in instrumental learning tasks [73]. Thus, stress appears to favour S-R learning strategies dependent on the striatum over prefrontal cortex-dependent goal-directed or hippocampus-dependent spatial strategy, respectively.

The studies discussed in this paper further show that while habit memory appears to be enhanced under stress, cognitive memory seems to be impaired [77, 78]. These distinct effects of stress on multiple memory systems were found using a single-solution spatial navigation task and they imply that striatum-dependent habit memory enhancement may even come at the expense of hippocampus-dependent cognitive memory. In line with this idea are the neuroimaging data from the study by Schwabe and Wolf [79], which revealed that the striatum gets significantly activated during a PCL task in both a stress and a control group, but that there was no significant activation of medial temporal lobe structures in the stress group. Importantly, left hippocampal activity was even negatively correlated with PCL performance in the stress group [79]. Since no differences in task performance were found between the stress and the control groups, it can be concluded that (1) a shift from cognitive to habit memory systems under stress can rescue task performance and (2) the attempt to engage the declarative memory system during the experience of stress even disrupts task performance. The interaction between the hippocampus-based memory system and the striatum-based memory system thus seems to be of a primarily competitive manner.

Investigating the role of the BLA concerning the shift from cognitive to habit memory, the results of the above-discussed studies indicate that the interaction of GCs and norepinephrine affects the engagement of multiple memory systems and the quantitative parameters of memory in a similar manner. Peripheral injections as well as intra-BLA infusions of the beta-adrenergic receptor antagonist RS 79948 impaired hippocampus-dependent memory and enhanced striatum-dependent memory [77, 78]. Similarly, many studies on quantitative memory parameters found that the process of memory retrieval under stress is impaired in spatial memory tasks [58, 59], as well as in declarative memory tasks [62–64], which may be explained by the fact that both spatial and declarative memory rely on the hippocampus. However, these studies on quantitative memory parameters report that the impairing effects of stress on spatial or declarative memory retrieval are abolished after blockage of beta-adrenergic receptors in the BLA, which leads to an increase in norepinephrine levels [60–64]. The same principle appears to be true for the engagement of multiple memory systems: higher norepinephrine levels due to injections of RS 79948 allow impairing effects on hippocampus-dependent spatial memory, whereas a simultaneous inactivation of the BLA abolishes this impairment [78]. These results suggest that the functional integrity of the BLA is of utter importance for stress to affect quantitative and qualitative parameters of hippocampus-based memory processes.

The behavioural tests which were developed for the study of multiple memory systems assess behavioural choices, which indicate the engagement of either procedural or declarative memory system in learning processes. Schwabe and Wolf [80] reported that the stress-induced use of the habit system can render participant's behaviour insensitive to changes in outcome value in an instrumental learning task. As discussed above, the quality of available information is critical for the process of cognitive appraisal of a situation. Taken together, the studies discussed in this review suggest that the quality of consolidated material is dependent on the engaged memory system during the learning process as well as during retrieval. If an S-R learning strategy is used and thus the striatum-dependant memory system is controlling the acquisition of a task, the subsequent behavioural choices have a habitual character [77, 78]. On the other hand, if a spatial strategy is used and thus the hippocampus-dependent declarative system is controlling consolidation processes, the subsequently retrieved information may come in the form of an (either true or false) internal representation of the external world. This cognitive model integrates and compares learned associations between multiple items and events and thus subsequent behavioural choices are based on a cognitive evaluation of the different available actions in respect of a desired outcome. Moreover, the discussed findings indicate that the memory system engaged in retrieval processes can modulate behaviour [80]. The retrieved information using a hippocampus-dependent strategy contains knowledge about action-outcome relationships which allows participants to choose goal-directed actions. A goal-directed behavioural strategy is dependent on different brain regions, including the prefrontal cortex, which also plays an important role in the cognitive appraisal of a situation. Moreover, coping with a stressor can increase cognitive load and thus limit available cognitive resources. If cognitive resources are reduced during the process of retrieval, the careful evaluation of hippocampus-based declarative memory is impaired [80]. Because under stress the prefrontal cortex seems to be already “busy” with a different task (coping), this brain area cannot be sufficiently engaged in the processes of retrieval and cognitive appraisal in order to enable the use of a goal-directed strategy. The subsequent switch to striatum-dependent habit system may result in actions, which do not lead to desired outcomes because the retrieved information only consists of S-R relationships and is thus insensitive to changes in the value of an outcome.

Interestingly, the S-R learning strategy refers to the procedural process of extracting common elements from a series of events, which results in the use of a heuristic rule-of-thumb in order to guide behaviour in a state of decreased cognitive resources. Behavioural choices on the basis of heuristic processing do not necessarily lead to adverse decisions but rather are adaptive mechanisms in order to operate effectively when cognitive load is high.

These theoretical considerations of the relationship between memory and behaviour may remind us of the dual-system theory proposed by Kahneman [82]. System 1, which is also referred to as intuition, results in a gut feeling regarding the current situation and can potentially bias people towards making irrational choices [82]. Moreover, system 1 operates in a parallel and automatic manner and is fast, effortless, and inflexible. All these characteristics are also attributable to the striatum-dependent habit memory system, which has been extensively discussed in this paper. On the other hand, the proposed system 2, which is also referred to as reasoning, operates in a controlled, slow, and serial manner and is effortful and flexible [82]. The hippocampus-dependant cognitive system as discussed in this paper thus seems to correspond to system 2. These similarities might be of valuable interest in the research on decision making. It is tempting to speculate that stress and emotional arousal can mediate the engagement of system 1 and system 2 in subsequent decision-making processes by modulating effects on multiple memory systems. It might thus be the case that the engagement of hippocampus-based cognitive and striatum-based habit memory during learning can determine subsequent behavioural choices because of qualitative aspects of retrieved information.

## 4. Conclusion

In mammal evolution, multiple memory systems have developed to serve different functions. On the one hand, incremental habit memory formation has evolved as a consequence of specialized performance systems which are reactivated in similar situations, thus allowing to react fast, albeit they are rather inflexible. On the other hand, a memory system has developed to serve everyday memory performance for unique episodes. Both systems are subserved by different neuroanatomical networks. As we have outlined in this review, the interaction between these memory systems is modulated by stress hormones: a physiological stress response leads to enhanced striatal habit memory and impaired hippocampal cognitive memory. Furthermore, we could show that the functional integrity of the amygdala is of utter importance in mediating this interaction, particularly concerning the impairing effects of stress on hippocampus-based memories.

These distinct memory systems are differentially involved in the learning process in different situations (e.g., stressful versus nonstressful) and help us to focus either on particular elements of a given situation in order to make quick decisions and “survive” (at the expense of overlooking some peripheral nonsalient details), or on the situation as a whole, in order to guide “rational” decision-making. Since the quality of consolidated memories depends on the engaged memory systems during learning as well as retrieval, it is argued that optimal memory functioning and decision-making require a balanced interaction between different neuroanatomical networks and their modulating stress hormones.

The aim of this contribution was to review and discuss the neural correlates of mammalian multiple memory systems and how they are affected by stress. We recapitulated the biological systems involved in a stress response, which are fundamental for the understanding of the herein discussed studies, on a macrolevel. However, before drawing conclusions, one should bear in mind that the behavioural and imaging results reported in our paper can only account for understanding part of the complex nature of memory. While the presented data helps pointing out this complexity, it certainly cannot fully explain it. Further research should focus on stress effects on a cellular level in order to gain a deeper understanding of the exact mechanisms responsible for the engagement of multiple memory systems. By integrating our knowledge concerning molecular and transcriptional alterations under stress into the herein proposed cognitive theories, we will hopefully be able to shed light onto the complex interactions between and within an organism's central and autonomic nervous systems and the endocrinological system during stressful experiences.

## 5. Overview of Presented Studies

See Table 1.

## Figures and Tables

**Table 1:** 

	Wingard and Packard [77]	Packard and Gabriele [78]	Schwabe and Wolf [80]	Schwabe and Wolf [79]
Task	Spatial navigation	Spatial navigation	Instrumental learning	Probabilistic Classification Learning (PCL)

Involved multiple memory systems	Hippocampus-dependent “cognitive” memory (place learning)	Hippocampus-dependent “cognitive” memory (place learning)	Goal-directed system (action-outcome learning): prefrontal cortex, dorsomedial thalamus, and dorsomedial striatum	Hippocampus-dependent declarative memory
	Dorsal striatum-dependent “habit” memory (response learning)	Dorsal striatum-dependent “habit” memory (response learning)	Habit system (S-R learning): dorsolateral striatum	Striatum-dependent procedural memory

Hypothesis	Intra-BLA administration of an anxiogenic biases rats towards the use of habit memory Intra-BLA infusion of RS-79948 is anxiogenic	Peripheral administration of an anxiogenic drug enhances and impairs response and place learning, respectively The functional integrity of the BLA may be critical for these effects	Acute stress favours habits over goal-directed actions when it is administered before the extinction test (after learning)	Stress may modulate the engagement of hippocampus-based declarative and striatum-based procedural memory systems in classification learning This may be observable in functional activity patterns assessed with fMRI

Sample	Rodents *n* = 59 male Charles River Long-Evans rats	Rodents *n* = 87 male Charles River Long-Evans rats	Humans *n* = 68 students (34 men, 34 women; 18–32 years) Exclusion of 17 subjects	Humans *n* = 60 students (30 men, 30 women; 18–30 years) Exclusion of 1 subject

Methods	Training (5 consecutive days, six trials/day) in single-solution water plus maze task (hippocampus-dependent place learning versus dorsal striatum-dependent response learning) Intra-BLA infusions of RS-79948 (place task *n* = 8, response task *n* = 7) or saline (place task *n* = 7, response task *n* = 7) immediately following training on days 1–3 Additional groups (place task *n* = 7, response task *n* = 7) received intra-BLA infusions of RS-79948 2 h after training Anxiogenic potential of RS-79948: standard anxiety test with an automated elevated plus maze apparatus after intra-BLA infusions of RS 79948 (*n* = 8) or saline (*n* = 8)	Training (6 consecutive days, six trials/day) in single-solution water plus maze task (hippocampus-dependent place learning versus dorsal striatum-dependent response learning) Infusions of either peripheral and intra-BLA saline (place task *n* = 7, response task *n* = 9), peripheral RS-79948 and intra-BLA saline (place task *n* = 9, response task *n* = 8), peripheral saline and intra-BLA bupivacaine (place task *n* = 7, response task *n* = 7), or peripheral RS 79948 and intra-BLA bupivacaine (place task *n* = 8, response task *n* = 7) immediately following training on days 1–3	Training in two instrumental actions (high versus low probability) leading to a food outcome: three randomized trial types with different outcomes: (i) Chocolate (75 trials) (ii) Orange (75 trials) (iii) Neutral (75 trials) Selective devaluation of one outcome (chocolate or orange) through eating to satiety Socially evaluated cold pressor test (SECPT) versus control condition Subjective stress ratings immediately after SECPT or control condition Extinction test with 75 trials of the three trial types (25 min after SECPT/control condition and approximately 40 min after training) Assessment of explicit task knowledge after extinction test (free recall and multiple-choice questionnaire) Hunger and pleasantness ratings before learning, before and after devaluation, and before the extinction test	SECPT/control condition before learning Subjective stress ratings immediately after SECPT or control condition Weather-prediction task (PCL task) and visuomotor control task (25 min after SECPT/control condition) in the scanner: 200 randomized trials (100 trials per task; 1 out of 14 different cue patterns is presented per trial) Assessment of explicit task knowledge (10 items questionnaire) outside the scanner Learning strategy analysis with a mathematical model (comparison of a participant's actual responses and the expected responses using a declarative or procedural strategy)

Induction of stress/arousal	BLA: injection of beta-adrenergic antagonist (RS 79948)	Peripheral injection of beta-adrenergic antagonist (RS 79948)	Socially evaluated cold pressure test (SECPT)	Socially evaluated cold pressure test (SECPT)

Physiological stress parameters	None measured	None measured	Blood pressure before, during, and after the SECPT or control condition Cortisol: saliva samples after arrival at the laboratory, just before, just after, and 20 and 50 min after the SECPT or control condition	Blood pressure before, during, and after the SECPT or control condition Cortisol: saliva samples before and immediately after the SECPT or control condition as well as before and after the learning task (25 and 90 min after the SECPT/control condition, resp.)

Affected memory phase	Consolidation	Consolidation	Retrieval	Encoding/consolidation

Behavioural results	Posttraining immediate intra-BLA infusions of RS-79948 (relative to delayed infusion or saline) impaired acquisition of the place task and enhanced acquisition of the response task Anxiogenic potential of RS 79948: intra-BLA infusions of RS 79948 (relative to saline) lead to more and less time spent in the closed and open arms of the maze, respectively	Posttraining peripheral RS 79948 injections enhance response learning BLA inactivation blocks the enhancement of response learning Posttraining peripheral RS 79948 injections impair place learning BLA inactivation blocks the impairment of place learning	SECPT increased subjective stress ratings Habitual behaviour after SECPT: participants indicated that they do not want the devalued outcome any more but still chose the referring action Goal-directed behaviour after control condition: control participants did not prefer the devalued food anymore; thus they did not prefer the associated action anymore No stress effects on explicit task knowledge	SECPT increased subjective stress ratings Comparable learning curves in the PCL task between SECPT/control condition: gradual improvement of classification performance across training Stress effects on learning strategy during PCL: decreased use of single-cue-based strategies (hippocampus-dependent) and increased use of multicue-based strategies (striatum-dependent) Stress effects on explicit task knowledge: participants in the SECPT condition remembered fewer details of the PCL task

Physiological results (stress parameters)	None measured	None measured	Increase in blood pressure during SECPT Increased salivary cortisol before extinction (20 min after SECPT) The SECPT-induced increase in cortisol levels (baseline-peak) is associated with habit performance	Increase in blood pressure during SECPT Systolic blood pressure during the SECPT/control condition correlates with the use of multicue-based strategies Increased salivary cortisol immediately before the PCL task in the scanner (25 min after SECPT) Salivary cortisol levels (across both groups and for all time points) correlate with PCL performance The SECPT-induced increase in cortisol levels (baseline-peak) is not associated with PCL performance Salivary cortisol levels 25 and 90 min after the SECPT correlate with the use of multicue-based strategies

Neuroimaging results	None	None	None	Activated brain areas during the PCL task: caudate nucleus, putamen, hippocampus, parahippocampal cortex, orbitofrontal cortex, cingulate cortex, and inferior frontal cortex Activity of the hippocampus correlated with the use of single-cue strategies, and activity of the putamen and the caudate nucleus correlated with multicue strategies during the PCL task Activation of the striatum during PCL in both groups, but no significant activation of medial temporal lobe structures in the stress group Caudate nucleus activity correlates with salivary cortisol levels (across both groups and for all time points), but not with the increase in cortisol levels (baseline-peak) after SECPT PCL performance in the stress group is positively correlated with activity in the right caudate nucleus and the left putamen, but negatively correlated with activity in the left hippocampus PCL performance in the control group is correlated with activity in the left hippocampus, but not with striatal activity

Conclusions	Intra-BLA infusions of RS 79948 can bias rats towards using habit memory by impairing cognitive memory Intra-BLA infusions of RS 79948 exert an anxiogenic effect at the same dose that impairs and enhances cognitive and habit memory, respectively Emotional state can modulate the degree of interference between cognitive and habit memory systems (release habit memory from competing/inhibitory influences of cognitive memory)	The functional integrity of the BLA is not necessary for the acquisition of place and response learning The functional integrity of the BLA is critical in order for peripheral injections of RS 79948 to impair hippocampus-dependent cognitive memory and enhance dorsal striatum-dependent habit memory, respectively	Acute stress before extinction testing can abolish sensitivity of performance to outcome value Acute stress can make behaviour habitual without affecting processes involved in learning (encoding, consolidation)	Stress does not affect the acquisition of the PCL task, but it changes the nature of classification learning from flexible, declarative learning to inflexible, procedural learning Stress impairs the hippocampus-dependent system and allows the striatum to control behaviour, which rescues task performance Attempts to engage the declarative system in PCL after stress disrupt performance

## References

[1] Packard M. G., Goodman J. (2012). Emotional arousal and multiple memory systems in the mammalian brain. *Frontiers in Behavioral Neuroscience*.

[2] Schwabe L., Wolf O. T. (2013). Stress and multiple memory systems: from 'thinking' to 'doing'.. *Trends in cognitive sciences*.

[3] De Kloet E. R., Derijk R. (2004). Signaling pathways in brain involved in predisposition and pathogenesis of stress-related disease: genetic and kinetic factors affecting the MR/GR balance. *Annals of the New York Academy of Sciences*.

[4] Lupien S. J., Oueliet-Morin I., Hupbach A., Cicchetti D., Cohen D. J. (2006). Beyond the stress concept: allostatic load—a developmental biological and cognitive perspective. *Developmental Psychopathology, Volume 2: Developmental Neuroscience*.

[5] Bao A. M., Meynen G., Swaab D. F. (2008). The stress system in depression and neurodegeneration: focus on the human hypothalamus. *Brain Research Reviews*.

[6] Sherin J. E., Nemeroff C. B. (2011). Post-traumatic stress disorder: the neurobiological impact of psychological trauma. *Dialogues in Clinical Neuroscience*.

[7] Reul J. M. H. M., de Kloet E. R. (1986). Anatomical resolution of two types of corticosterone receptor sites in rat brain with in vitro autoradiography and computerized image analysis. *Journal of Steroid Biochemistry*.

[8] Roozendaal B. (2002). Stress and memory: opposing effects of glucocorticoids on memory consolidation and memory retrieval. *Neurobiology of Learning and Memory*.

[9] McIntyre C. K., McGaugh J. L., Williams C. L. (2012). Interacting brain systems modulate memory consolidation. *Neuroscience and Biobehavioral Reviews*.

[10] Schreurs J., Seelig T., Schulman H. (1986). *β*
_2_-adrenergic receptors on peripheral nerves. *Journal of Neurochemistry*.

[11] Lawrence A. J., Watkins D., Jarrott B. (1995). Visualization of beta-adrenoceptor binding sites on human inferior vagal ganglia and their axonal transport along the rat vagus nerve. *Journal of Hypertension*.

[12] Kalia M., Sullivan J. M. (1982). Brainstem projections of sensory and motor components of the vagus nerve in the rat. *Journal of Comparative Neurology*.

[13] Sumal K. K., Blessing W. W., Joh T. H., Reis D. J., Pickel V. M. (1983). Synaptic interaction of vagal afferents and catecholaminergic neurons in the rat nucleus tractus solitarius. *Brain Research*.

[14] Van Bockstaele E. J., Peoples J., Telegan P. (1999). Efferent projections of the nucleus of the solitary tract to peri-locus coeruleus dendrites in rat brain: evidence for a monosynaptic pathway. *Journal of Comparative Neurology*.

[15] Asan E. (1998). *The Catecholaminergic Innervation of the Rat Amygdala*.

[16] Fallon J. H., Koziell D. A., Moore R. Y. (1978). Catecholamine innervation of the basal forebrain. II. Amygdala, suprarhinal cortex and entorhinal cortex. *Journal of Comparative Neurology*.

[17] Loughlin S. E., Foote S. L., Bloom F. E. (1986). Efferent projections of nucleus locus coeruleus: topographic organization of cells of origin demonstrated by three-dimensional reconstruction. *Neuroscience*.

[18] Ricardo J. A., Koh E. T. (1978). Anatomical evidence of direct projections from the nucleus of the solitary tract to the hypothalamus, amygdala, and other forebrain structures in the rat. *Brain Research*.

[19] Abercrombie H. C., Chambers A. S., Greischar L., Monticelli R. M. (2008). Orienting, emotion, and memory: phasic and tonic variation in heart rate predicts memory for emotional pictures in men. *Neurobiology of Learning and Memory*.

[20] Anderson A. K., Yamaguchi Y., Grabski W., Lacka D. (2006). Emotional memories are not all created equal: evidence for selective memory enhancement. *Learning & Memory*.

[21] Cahill L., Alkire M. T. (2003). Epinephrine enhancement of human memory consolidation: interaction with arousal at encoding. *Neurobiology of Learning and Memory*.

[22] Cahill L., Prins B., Weber M., McGaugh J. L. (1994). *β*-Adrenergic activation and memory for emotional events. *Nature*.

[23] Critchley H. D., Mathias C. J., Dolan R. J. (2002). Fear conditioning in humans: the influence of awareness and autonomic arousal on functional neuroanatomy. *Neuron*.

[24] Moor T., Mundorff L., Bohringer A. (2005). Evidence that baroreflex feedback influences long-term incidental visual memory in men. *Neurobiology of Learning and Memory*.

[25] Nielson K. A., Yee D., Erickson K. I. (2005). Memory enhancement by a semantically unrelated emotional arousal source induced after learning. *Neurobiology of Learning and Memory*.

[26] Kempermann G. (2012). New neurons for ‘survival of the fittest’. *Nature Reviews Neuroscience*.

[27] Snyder J. S., Soumier A., Brewer M., Pickel J., Cameron H. A. (2011). Adult hippocampal neurogenesis buffers stress responses and depressive behaviour. *Nature*.

[28] Surget A., Tanti A., Leonardo E. D. (2011). Antidepressants recruit new neurons to improve stress response regulation. *Molecular Psychiatry*.

[29] Anacker C., Cattaneo A., Luoni A. (2013). Glucocorticoid-related molecular signaling pathways regulating hippocampal neurogenesis. *Neuropsychopharmacology*.

[30] Renault V. M., Rafalski V. A., Morgan A. A. (2009). FoxO_3_ regulates neural stem cell homeostasis. *Cell Stem Cell*.

[31] Wu Y., Peng H., Cui M., Whitney N. P., Huang Y., Zheng J. C. (2009). CXCL12 increases human neural progenitor cell proliferation through Akt-1/FOXO3a signaling pathway. *Journal of Neurochemistry*.

[32] Graciarena M., Depino A. M., Pitossi F. J. (2010). Prenatal inflammation impairs adult neurogenesis and memory related behavior through persistent hippocampal TGFbeta1 downregulation. *Brain, Behavior, and Immunity*.

[33] Ahn S., Joyner A. L. (2005). In vivo analysis of quiescent adult neural stem cells responding to Sonic hedgehog. *Nature*.

[34] He Y., Zhang H., Yung A. (2014). ALK5-dependent TGF-*β* 2 signaling is a major determinant of late-stage adult neurogenesis. *Nature Neuroscience*.

[35] Johnson J. D., Campisi J., Sharkey C. M. (2005). Catecholamines mediate stress-induced increases in peripheral and central inflammatory cytokines. *Neuroscience*.

[36] Mahar I., Bambico F. R., Mechawar N., Nobrega J. N. (2014). Stress, serotonin, and hippocampal neurogenesis in relation to depression and antidepressant effects. *Neuroscience and Biobehavioral Reviews*.

[37] Squire L. R. (2004). Memory systems of the brain: a brief history and current perspective. *Neurobiology of Learning and Memory*.

[38] Tulving E. (1983). *Elements of Episodic Memory*.

[39] Shimamura A. P., Squire L. R. (1987). A neuropsychological study of fact memory and source amnesia. *Journal of Experimental Psychology: Learning, Memory, and Cognition*.

[40] Tulving E. (1989). Remembering and knowing the past. *American Scientist*.

[41] Kim J. J., Baxter M. G. (2001). Multiple brain-memory systems: the whole does not equal the sum of its parts. *Trends in Neurosciences*.

[42] Packard M. G., Hirsh R., White N. M. (1989). Differential effects of fornix and caudate nucleus lesions on two radial maze tasks: evidence for multiple memory systems. *The Journal of Neuroscience*.

[43] Joëls M., Pu Z., Wiegert O., Oitzl M. S., Krugers H. J. (2006). Learning under stress: how does it work?. *Trends in Cognitive Sciences*.

[44] Nater U. M., Moor C., Okere U. (2007). Performance on a declarative memory task is better in high than low cortisol responders to psychosocial stress. *Psychoneuroendocrinology*.

[45] Schwabe L., Bohringer A., Chatterjee M., Schachinger H. (2008). Effects of pre-learning stress on memory for neutral, positive and negative words: different roles of cortisol and autonomic arousal. *Neurobiology of Learning and Memory*.

[46] Elzinga B. M., Bakker A., Bremner J. D. (2005). Stress-induced cortisol elevations are associated with impaired delayed, but not immediate recall. *Psychiatry Research*.

[47] Kim J. J., Lee H. J., Han J.-S., Packard M. G. (2001). Amygdala is critical for stress-induced modulation of hippocampal long-term potentiation and learning. *The Journal of Neuroscience*.

[48] Kirschbaum C., Wolf O. T., May M., Wippich W., Hellhammer D. H. (1996). Stress- and treatment-induced elevations of cortisol levels associated with impaired declarative memory in healthy adults. *Life Sciences*.

[49] Lupien S. J., Gaudreau S., Tchiteya B. M. (1997). Stress-induced declarative memory impairment in healthy elderly subjects: relationship to cortisol reactivity. *Journal of Clinical Endocrinology and Metabolism*.

[50] Payne J. D., Jackson E. D., Ryan L., Hoscheidt S., Jacobs W. J., Nadel L. (2006). The impact of stress on neutral and emotional aspects of episodic memory. *Memory*.

[51] Tops M., van der Pompe G., Baas D. (2003). Acute cortisol effects on immediate free recall and recognition of nouns depend on stimulus valence. *Psychophysiology*.

[52] Roozendaal B., McGaugh J. L. (1997). Basolateral amygdala lesions block the memory-enhancing effect of glucocorticoid administration in the dorsal hippocampus of rats. *European Journal of Neuroscience*.

[53] Roozendaal B., Okuda S., Van Der Zee E. A., McGaugh J. L. (2006). Glucocorticoid enhancement of memory requires arousal-induced noradrenergic activation in the basolateral amygdala. *Proceedings of the National Academy of Sciences of the United States of America*.

[54] Oitzl M. S., de Kloet E. R. (1992). Selective corticosteroid antagonists modulate specific aspects of spatial orientation learning. *Behavioral Neuroscience*.

[55] Sandi C., Rose S. P. R. (1994). Corticosteroid receptor antagonists are amnestic for passive avoidance learning in day-old chicks. *European Journal of Neuroscience*.

[56] Roozendaal B., McGaugh J. L. (1996). Amygdaloid nuclei lesions differentially affect glucocorticoid-induced memory enhancement in an inhibitory avoidance task. *Neurobiology of Learning and Memory*.

[57] Abercrombie H. C., Speck N. S., Monticelli R. M. (2006). Endogenous cortisol elevations are related to memory facilitation only in individuals who are emotionally aroused. *Psychoneuroendocrinology*.

[58] de Quervain D. J.-F., Roozendaal B., McGaugh J. L. (1998). Stress and glucocorticoids impair retrieval of long-term spatial memory. *Nature*.

[59] Diamond D. M., Campbell A. M., Park C. R. (2006). Influence of predator stress on the consolidation versus retrieval of long-term spatial memory and hippocampal spinogenesis. *Hippocampus*.

[60] Roozendaal B., Griffith Q. K., Buranday J., De Quervain D. J.-F., McGaugh J. L. (2003). The hippocampus mediates glucocorticoid-induced impairment of spatial memory retrieval: dependence on the basolateral amygdala. *Proceedings of the National Academy of Sciences of the United States of America*.

[61] Roozendaal B., Hahn E. L., Nathan S. V., De Quervain D. J.-F., McGaugh J. L. (2004). Glucocorticoid effects on memory retrieval require concurrent noradrenergic activity in the hippocampus and basolateral amygdala. *Journal of Neuroscience*.

[62] Kuhlmann S., Wolf O. T. (2006). A non-arousing test situation abolishes the impairing effects of cortisol on delayed memory retrieval in healthy women. *Neuroscience Letters*.

[63] de Quervain D. J.-F., Aerni A., Roozendaal B. (2007). Preventive effect of *β*-adrenoceptor blockade on glucocorticoid-induced memory retrieval deficits. *The American Journal of Psychiatry*.

[64] Schwabe L., Oitzl M. S., Richter S., Schächinger H. (2009). Modulation of spatial and stimulus-response learning strategies by exogenous cortisol in healthy young women. *Psychoneuroendocrinology*.

[65] Buchanan T. W., Tranel D. (2008). Stress and emotional memory retrieval: effects of sex and cortisol response. *Neurobiology of Learning and Memory*.

[66] Domes G., Rothfischer J., Reichwald U., Hautzinger M. (2005). Inverted-U function between salivary cortisol and retrieval of verbal memory after hydrocortisone treatment. *Behavioral Neuroscience*.

[67] Schwabe L., Römer S., Richter S., Dockendorf S., Bilak B., Schächinger H. (2009). Stress effects on declarative memory retrieval are blocked by a beta-adrenoceptor antagonist in humans. *Psychoneuroendocrinology*.

[68] de Kloet E. R., Oitzl M. S., Joëls M. (1999). Stress and cognition: are corticosteroids good or bad guys?. *Trends in Neurosciences*.

[69] Diamond D. M., Campbell A. M., Park C. R., Halonen J., Zoladz P. R. (2007). The temporal dynamics model of emotional memory processing: a synthesis on the neurobiological basis of stress-induced amnesia, flashbulb and traumatic memories, and the Yerkes-Dodson law. *Neural Plasticity*.

[70] Packard M. G., Wingard J. C. (2004). Amygdala and ‘emotional’ modulation of the relative use of multiple memory systems. *Neurobiology of Learning and Memory*.

[71] Elliott A. E., Packard M. G. (2008). Intra-amygdala anxiogenic drug infusion prior to retrieval biases rats towards the use of habit memory. *Neurobiology of Learning and Memory*.

[72] Schwabe L., Oitzl M. S., Philippsen C. (2007). Stress modulates the use of spatial versus stimulus-response learning strategies in humans. *Learning and Memory*.

[73] Schwabe L., Wolf O. T. (2009). Stress prompts habit behavior in humans. *The Journal of Neuroscience*.

[74] Schwabe L., Dalm S., Schächinger H., Oitzl M. S. (2008). Chronic stress modulates the use of spatial and stimulus-response learning strategies in mice and man. *Neurobiology of Learning and Memory*.

[75] Dias-Ferreira E., Sousa J. C., Melo I. (2009). Chronic stress causes frontostriatal reorganization and affects decision-making. *Science*.

[76] Vyas A., Mitra R., Rao B. S. S., Chattarji S. (2002). Chronic stress induces contrasting patterns of dendritic remodeling in hippocampal and amygdaloid neurons. *The Journal of Neuroscience*.

[77] Wingard J. C., Packard M. G. (2008). The amygdala and emotional modulation of competition between cognitive and habit memory. *Behavioural Brain Research*.

[78] Packard M. G., Gabriele A. (2009). Peripheral anxiogenic drug injections differentially affect cognitive and habit memory: role of basolateral amygdala. *Neuroscience*.

[79] Schwabe L., Wolf O. T. (2012). Stress modulates the engagement of multiple memory systems in classification learning. *Journal of Neuroscience*.

[80] Schwabe L., Wolf O. T. (2010). Socially evaluated cold pressor stress after instrumental learning favors habits over goal-directed action. *Psychoneuroendocrinology*.

[81] Roozendaal B., McEwen B. S., Chattarji S. (2009). Stress, memory and the amygdala. *Nature Reviews Neuroscience*.

[82] Kahneman D., Frangsmyr T. (2003). Maps of bounded rationality: a perspective on intuitive judgement and choice. *Les Prix Nobel: The Nobel Prizes 2002*.

